# Treatment Modifications in Acute Coronary Syndrome Patients Treated with Ticagrelor: Insights from the FORCE-ACS Registry

**DOI:** 10.1055/a-2421-8866

**Published:** 2024-10-29

**Authors:** Niels M.R. van der Sangen, Jaouad Azzahhafi, Dean R.P.P. Chan Pin Yin, Lucas J.G. Zaaijer, Wout W.A. van den Broek, Ronald J. Walhout, Melvyn Tjon Joe Gin, Ron Pisters, Deborah M. Nicastia, Jorina Langerveld, Georgios J. Vlachojannis, Rutger J. van Bommel, Yolande Appelman, José P.S. Henriques, Wouter J. Kikkert, Jurriën M. ten Berg

**Affiliations:** 1 Department of Cardiology, Amsterdam UMC, University of Amsterdam, Amsterdam Cardiovascular Sciences, Amsterdam, The Netherlands; 2 Department of Cardiology, St. Antonius Hospital, Nieuwegein, The Netherlands; 3 Department of Cardiology, Hospital Gelderse Vallei, Ede, The Netherlands; 4 Department of Cardiology, Rijnstate Hospital, Arnhem, The Netherlands; 5 Department of Cardiology, Gelre Hospitals, Apeldoorn, The Netherlands; 6 Department of Cardiology, Rivierenland Hospital, Tiel, The Netherlands; 7 Department of Cardiology, University Medical Center Utrecht, Utrecht, The Netherlands; 8 Department of Cardiology, Tergooi Hospital, Hilversum, The Netherlands; 9 Department of Cardiology, Amsterdam UMC, VU University, Amsterdam Cardiovascular Sciences, Amsterdam, The Netherlands; 10 Department of Cardiology, University Medical Center Maastricht, Maastricht, The Netherlands

**Keywords:** acute coronary syndrome, treatment modifications, ticagrelor

## Abstract

**Aims:**

Patients presenting with acute coronary syndrome (ACS) are frequently treated with the P2Y _12_ -inhibitor ticagrelor. Some patients prematurely discontinue ticagrelor, but the incidence of reasons for and clinical implications of treatment modification are relatively unknown.

**Methods and Results:**

Data from 4,278 ACS patients (mean age: 63.6 years, 26.1% women) who were discharged on ticagrelor and enrolled in the FORCE-ACS registry between 2015 and 2020 were used. Treatment modifications were categorized as physician-recommended discontinuation, alteration, interruption, or disruption and occurred in 26.7, 20.1, 2.8, and 3.1% of patients within 12 months of follow-up ( **Visual Summary** ). Underlying reasons for treatment modification differed per type of modification. Overall, the rate of ischemic events defined as all-cause death, myocardial infarction, or stroke was 6.6% at 12 months of follow-up. Cox regression analysis using time-updated modification variables as independent variables showed that treatment interruption (adjusted hazard ratio [HR]: 2.93, 95% confidence interval [CI]: 1.48–5.79, *p* < 0.01) and disruption (adjusted HR: 2.33, 95% CI: 1.07–5.07, *p* = 0.03) were associated with an increased risk of ischemic events even after adjustment for relevant confounders. Discontinuation and alteration were not associated with increased ischemic risk.

**Conclusion:**

In clinical practice, treatment modifications in ACS patients discharged on ticagrelor are common, although type and reasons for modification are heterogeneous. Treatment interruption and disruption are associated with excess cardiovascular risk.

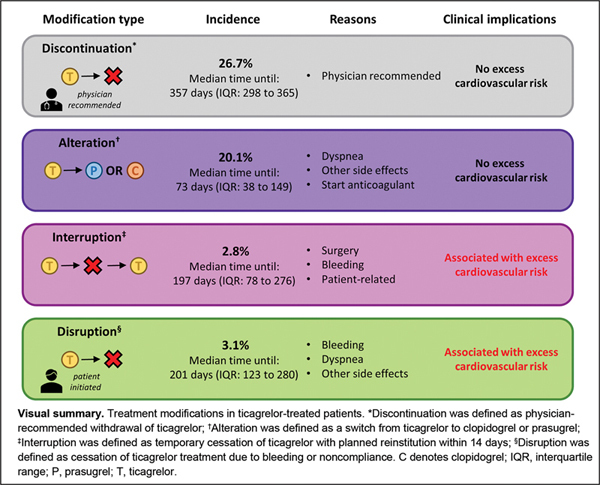

## Introduction

Patients presenting with acute coronary syndrome (ACS) are frequently treated with potent P2Y _12_ -inhibitors, such as ticagrelor. 1 Ticagrelor reversibly binds to the P2Y _12_ -receptor and as a result prevents platelet activation and aggregation. 2 The antiplatelet activity of ticagrelor is not reliant on metabolic activation and ticagrelor therefore has a more rapid and predictable effect compared with clopidogrel. 3 In the double-blind, randomized Platelet Inhibition and Patient Outcomes (PLATO) trial, ticagrelor significantly reduced the composite of cardiovascular death, myocardial infarction (MI), or stroke compared with clopidogrel in patients presenting with ACS. 4 Ever since, ACS patients are frequently treated with ticagrelor on top of aspirin (i.e., dual antiplatelet therapy [DAPT]) for at least 12 months. 1 However, not all patients tolerate ticagrelor. Common side effects include bleeding and dyspnea, and although these side effects are usually mild and transient, some patients require treatment modification within 12 months. 5 Clinical reasons and underlying context for treatment modification are heterogeneous, and it is unclear what, if any, excess cardiovascular risk can be attributed to these modifications. Previous studies have mostly classified patients using a binary, on-versus-off treatment approach, whereas differentiation in type and reason for modification might be important. 6 7 Therefore, using real-world data from the FORCE-ACS registry, our study aimed to examine (1) the incidence of, (2) reasons for, and (3) clinical implications of treatment modifications in patients discharged on ticagrelor.

## Methods

### Study Design and Patient Population

The rationale and design of the FORCE-ACS registry have been described previously. 8 In short, the FORCE-ACS registry is an ongoing prospective registry of nine Dutch hospitals. The primary aim of the registry is to provide insight into different aspects of the diagnosis, management, and follow-up of patients with ACS. From 2015 onward, all consecutive adult patients admitted for (suspected) ACS were eligible for participation. For the present study, all patients who were discharged with an active ticagrelor prescription after their initial hospital admission were included. Patients were treated with ticagrelor 90 mg twice daily following a loading dose of 180 mg in line with current guidelines. 1 The institutional review boards of the participating centers approved the protocol of the FORCE-ACS registry, and written informed consent was obtained from each patient. The present study complies with the principles of the Declaration of Helsinki and reports according to the STrengthening the Reporting of OBservational studies in Epidemiology statement. 9

### Definitions

Treatment modifications were classified as discontinuation, alteration, interruption, or disruption. For discontinuation, interruption and disruption definitions previously set out by Mehran et al in the patterns of nonadherence to antiplatelet regimens in stented patients (PARIS) registry were used. 6 Hence, discontinuation was defined as physician-recommended withdrawal of ticagrelor for patients thought to no longer need ticagrelor. Alteration was defined as a switch from ticagrelor to clopidogrel or prasugrel. Interruption was defined as temporary cessation of ticagrelor, for example, due to surgical necessity, with planned reinstitution within 14 days and disruption was defined as cessation of ticagrelor treatment due to bleeding or noncompliance. The primary ischemic endpoint was time till the first occurrence of all-cause death, MI, or stroke. MI and stroke were included in the primary ischemic endpoint regardless of etiology. MI was classified according to the 4 ^th^ universal definition of MI, which includes stent thrombosis (MI type 4b). 10

### Follow-up

Treatment modifications and clinical events were reported via questionnaires at 1 and 12 months after hospital admission. If patients did not complete the questionnaires, they were contacted by phone. Additionally, the electronic health records of all patients were checked. In case of treatment modification and/or a clinical event, relevant source document was collected and patients were asked to provide information about the date of and reason for treatment modification. Information regarding treatment modification was corroborated by prescription data reported by the pharmacy. Treatment modifications and clinical events were reviewed and adjudicated by the first two authors who had full access to the patient's electronic health record.

### Statistical Analysis

Continuous variables were reported as mean ± standard deviation or median with interquartile range (IQR) as appropriate and categorical variables were reported as frequencies and percentages. Patient characteristics were compared by modification status using an independent *t* -test for continuous variables and a chi-square test or a Fisher's exact test for categorical variables using patients without treatment modification as a reference. Since patients could have more than one type of treatment modification, patients were grouped according to the most severe type of modification (disruption was considered the most severe type of modification followed by interruption, alteration, and discontinuation). Clinical implications of treatment modifications were assessed using the Cox regression models using time-updated modification variables as independent variable. The patient's follow-up time was broken into time periods spent in each modification type based on the previously described hierarchy (i.e., patients were only reclassified if a more severe treatment modification occurred). All models were adjusted for the following potential confounders: age, sex, initial diagnosis (i.e., unstable angina, non-ST-segment elevation myocardial infarction [NSTEMI] or ST-segment elevation myocardial infarction [STEMI]), revascularization during initial hospital admission (i.e., percutaneous coronary intervention [PCI] or coronary artery bypass grafting [CABG]) and presence of at least one concomitant chronic disease (i.e., diabetes, atrial fibrillation, chronic kidney disease, chronic obstructive pulmonary disease, or peripheral artery disease). Potential confounders were selected based on literature, clinical judgement, and availability during hospital admission. In case of treatment modification on the same day as an ischemic event, the model took into account the exact moment of treatment modification (i.e., before or after the ischemic event). Results are presented as hazard ratio (HR) with corresponding 95% confidence interval (CI) per modification type. Significance was set at a *p* -value of <0.05. Statistical analyses were performed using SPSS version 28 (SPSS Inc., Chicago, Illinois, United States) and illustrative graphics were composed using GraphPad Prism version 8.3 (GraphPad Software, San Diego, California, United States).

## Results

### Patient Characteristics

**Fig. 1:**
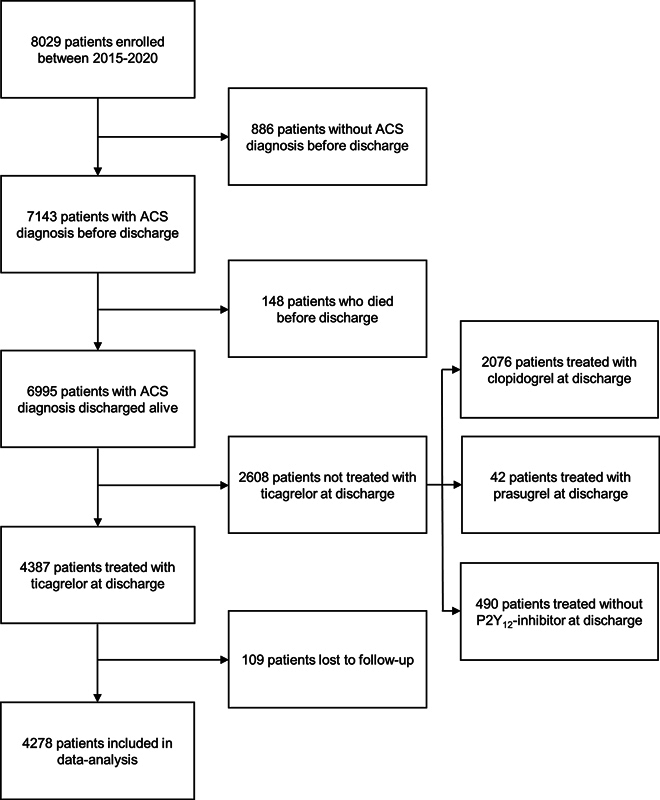
Flowchart. ACS, acute coronary syndrome.

From January 2015 until December 2020, 8,029 patients were included in the FORCE-ACS registry. Patients who were ultimately not diagnosed with ACS ( *n* = 886) or who did not survive the index hospital admission ( *n* = 148) were excluded. In total, 4,387 out of 6,995 patients (62.7%) were discharged on ticagrelor, whereas 2,076 patients (29.7%) were treated with clopidogrel, 42 patients (0.6%) with prasugrel, and 490 patients (7.0%) without a P2Y _12_ -inhibitor. Patients ( *n* = 109) who did not complete 12 months of follow-up were excluded. Hence, 4,278 patients were included in the present analysis. A detailed flowchart is provided in Fig. 1 .

The mean age at time of enrollment was 63.6 ± 11.5 years and 26.1% of patients were female ( Table 1 ). Overall, 5.2, 46.4, and 48.5% of patients were diagnosed with unstable angina, NSTEMI and STEMI, respectively. Almost all patients (98.3%) underwent coronary angiography during initial hospital admission and 80.1 and 7.3% of patients subsequently underwent PCI or CABG ( Table 2 ). Per design, all patients included in the present analysis were treated with ticagrelor at time of discharge, whereas 98.0% of these patients were also treated with aspirin. Moreover, 76.0, 78.1, and 95.6% of patients were treated with a β-blocker, an angiotensin-converting enzyme inhibitor or angiotensin receptor blocker and at least one cholesterol-lowering drug (e.g., statin, ezetimibe, or a PSCK9-inhibitor). Based on the PRECISE-DAPT and DAPT scores, 19.7 and 41.4% of patients were considered at high bleeding risk (defined as a PRECISE-DAPT score ≥ 25) and high ischemic risk (defined as a DAPT score ≥ 2), respectively.

**Table 1:** Baseline characteristics of patients with and without treatment modification

	No modification	Discontinuation		Alteration		Interruption		Disruption	
	*n* = 2,312	*n* = 910	*p* -Value	*n* = 814	*p* -Value	*n* = 111	*p* -Value	*n* = 131	*p* -Value
Age (y)	62.6 ± 11.5	64.0 ± 11.6	<0.01	64.9 ± 11.2	<0.01	67.0 ± 11.0	<0.01	66.8 ± 11.8	<0.01
Body mass index (kg/m ^2^ ) a	27.6 ± 4.4	27.2 ± 4.3	0.02	27.8 ± 4.8	0.28	27.6 ± 4.0	0.89	27.9 ± 4.4	0.53
Female sex (%)	559 (24.2%)	230 (25.3%)	0.52	250 (30.7%)	<0.01	27 (24.3%)	0.97	49 (37.4%)	<0.01
Current or former smokers (%)	1,403 (60.7%)	539 (59.3%)	0.46	519 (63.8%)	0.03	72 (64.8%)	0.16	85 (64.8%)	0.05
Hypertension (%)	1,127 (48.7%)	420 (46.2%)	<0.01	420 (51.6%)	0.37	67 (60.4%)	0.04	74 (56.5%)	0.23
Hyperlipidemia (%)	1,191 (51.5%)	450 (49.6%)	0.02	436 (53.6%)	0.46	57 (51.4%)	0.99	74 (56.5%)	0.13
Diabetes mellitus (%)	387 (16.7%)	123 (13.5%)	0.02	159 (19.6%)	0.12	33 (29.7%)	<0.01	25 (19.1%)	0.57
Chronic kidney disease (%) b	274 (11.9%)	117 (12.9%)	0.43	116 (14.3%)	0.08	21 (18.9%)	0.03	16 (12.2%)	0.90
COPD	136 (5.9%)	52 (5.7%)	0.86	61 (7.5%)	0.10	14 (12.6%)	<0.01	13 (9.9%)	0.06
Peripheral artery disease	102 (4.4%)	33 (3.6%)	0.32	58 (7.1%)	<0.01	12 (10.8%)	<0.01	9 (6.9%)	0.19
Atrial fibrillation (%)	30 (1.3%)	11 (1.2%)	0.84	21 (2.6%)	0.01	1 (0.9%)	0.72	3 (2.3%)	0.34
Prior stroke or TIA (%)	112 (4.8%)	43 (4.7%)	0.89	61 (7.5%)	<0.01	7 (6.3%)	0.47	7 (5.3%)	0.80
Prior MI (%)	386 (16.8%)	125 (13.7%)	0.04	163 (20.0%)	0.03	25 (22.9%)	0.10	28 (21.5%)	0.16
Prior PCI (%)	386 (16.8%)	132 (14.6%)	0.12	175 (21.6)	<0.01	29 (26.4%)	<0.01	26 (20.0%)	0.34
Prior CABG (%)	106 (4.6%)	29 (3.2%)	0.08	62 (7.6%)	<0.01	10 (9.0%)	0.03	8 (6.1%)	0.42
Cardiac arrest at admission (%)	98 (4.2%)	31 (3.4%)	0.55	17 (2.1%)	0.02	3 (2.7%)	0.68	3 (2.3%)	0.51
Killip class (%) I II III IV	2,094 (90.7%) 163 (7.1%) 5 (0.2%) 22 (1.0%)	831 (91.8%) 57 (6.3%) 0 (0.0%) 9 (1.0%)	0.60	731 (89.9%) 63 (7.7%) 2 (0.2%) 5 (0.6%)	0.69	93 (83.8%) 15 (13.5%) 0 (0.0%) 2 (1.8%)	0.39	118 (90.1%) 9 (6.9%) 0 (0.0%) 1 (0.8%)	0.28
Clinical diagnosis (%) Unstable angina NSTEMI STEMI	103 (4.5%) 1,027 (44.4%) 1,182 (51.1%)	35 (3.8%) 414 (45.5%) 461 (50.7%)	0.68	59 (7.2%) 408 (50.1%) 347 (42.6%)	<0.01	12 (10.8%) 61 (55.0%) 38 (34.2%)	<0.01	12 (9.2%) 73 (55.7%) 46 (35.1%)	<0.01
PRECISE-DAPT score ≥ 25 (%)	402 (17.4%)	208 (22.9%)	<0.01	167 (20.5%)	0.05	29 (26.1%)	0.02	38 (29.0%)	<0.01
DAPT score ≥ 2 (%)	1,013 (43.8%)	335 (36.8%)	<0.01	334 (41.0%)	0.15	47 (42.3%)	0.67	42 (32.1%)	0.01

Abbreviations: CABG, coronary artery bypass grafting; COPD, chronic obstructive pulmonary disease; MI, myocardial infarction; NSTEMI, non-ST-segment elevation myocardial infarction; PCI, percutaneous coronary intervention; STEMI, ST-segment elevation myocardial infarction; TIA, transient ischemic attack.

Note: Values are presented as mean ± standard deviation or number of patients (%).

a Body mass index was missing in 237 cases (5.5%).

b Chronic kidney disease was defined as a glomerular filtration rate < 60 mL/min/1.73 m ^2^ .

**Table 2:** In-hospital management of patients with and without treatment modification

	No modification	Discontinuation		Alteration		Interruption		Disruption	
	*n* = 2,312	*n* = 910	*p* -Value	*n* = 814	*p* -Value	*n* = 111	*p* -Value	*n* = 131	*p* -Value
Coronary angiography (%)	2,281 (98.7%)	888 (97.6%)	0.03	805 (98.9%)	0.61	109 (98.2%)	0.66	122 (93.1%)	<0.01
PCI (%)	1,945 (84.6%)	683 (75.1%)	<0.01	645 (79.2%)	<0.01	82 (74.5%)	<0.01	71 (55.9%)	<0.01
CABG (%)	148 (6.4%)	77 (8.5%)	0.04	57 (7.0%)	0.55	11 (10.0%)	0.14	18 (13.7%)	<0.01
Aspirin (%)	2,270 (98.2%)	892 (98.0%)	0.76	794 (97.5%)	0.26	109 (98.2%)	0.99	127 (96.9%)	0.31
Oral anticoagulants (%)	57 (2.5%)	29 (3.2%)	0.25	25 (3.1%)	0.35	3 (2.7%)	0.88	5 (3.8%)	0.34
Beta-blocker (%)	1,784 (77.2%)	681 (74.8%)	0.16	606 (74.4%)	0.12	87 (78.4%)	0.77	95 (72.5%)	0.22
ACE-inhibitor or ARB (%)	1,835 (79.4%)	712 (78.2%)	0.48	621 (76.3%)	0.07	82 (73.9%)	0.16	93 (71.0%)	0.02
Cholesterol lowering-drugs (%)	2,214 (95.8%)	867 (95.3%)	0.54	779 (95.7%)	0.94	109 (98.2%)	0.21	120 (91.6%)	0.03

Abbreviations: ACE, angiotensin-converting enzyme; ARB, angiotensin receptor blocker; CABG, coronary artery bypass grafting; PCI, percutaneous coronary intervention.

Note: Values are presented as number of patients (%).

### Treatment Modifications

**Fig. 2:**
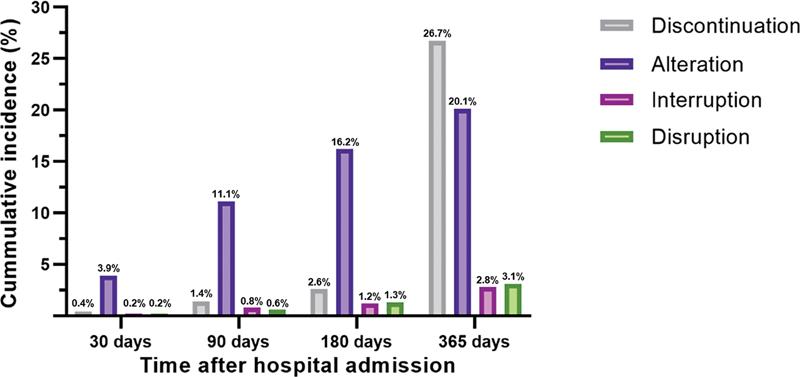
Cumulative incidence of treatment modification. Note that individual patients could have more than one type of treatment modification.

**Fig. 3:**
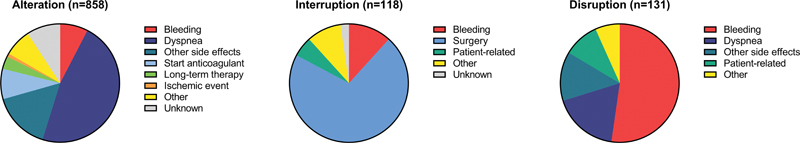
Underlying reasons for treatment modification. Note that individual patients could have more than one type of treatment modification. Sample size ( *n* ) refers to number of individual patients for each type of modification.

The cumulative incidence of treatment modifications during the first 12 months of follow-up is shown in Fig. 2 . Reasons for treatment modification are shown in Fig. 3 . Most treatment modifications were physician-recommended discontinuation of ticagrelor after the intended treatment duration was completed (26.7%). Median time until discontinuation was 357 days (IQR: 298–365). Rates for alteration, interruption, and disruption were 20.1, 2.8, and 3.1%, respectively. Alteration most often occurred before 6 months (median time until alteration 73 days [IQR 38–149]) and common reasons were dyspnea (47.2%), other side effects (15.8%), and a new indication for oral anticoagulation (8.3%). Alteration due to bleeding (7.6%) or new ischemic events (0.8%) was less common. Most patients with an alteration due to bleeding switched from ticagrelor to clopidogrel (98.6%) and only 1.4% to prasugrel. Patients with an alteration for other reasons switched to clopidogrel and prasugrel in 80.6% and 19.4% of cases, respectively. Interruption was primarily due to surgical necessity (71.1%). The most common reasons for disruption were bleeding (52.2%) and dyspnea (17.9%). Interruption and disruption most frequently occurred between 6 and 12 months after initial hospital admission. Most patients (38.4%) had only one treatment modification during follow-up, and 324 patients (7.6%) had two or more modifications.

### Clinical Implications of Modification

**Fig. 4:**
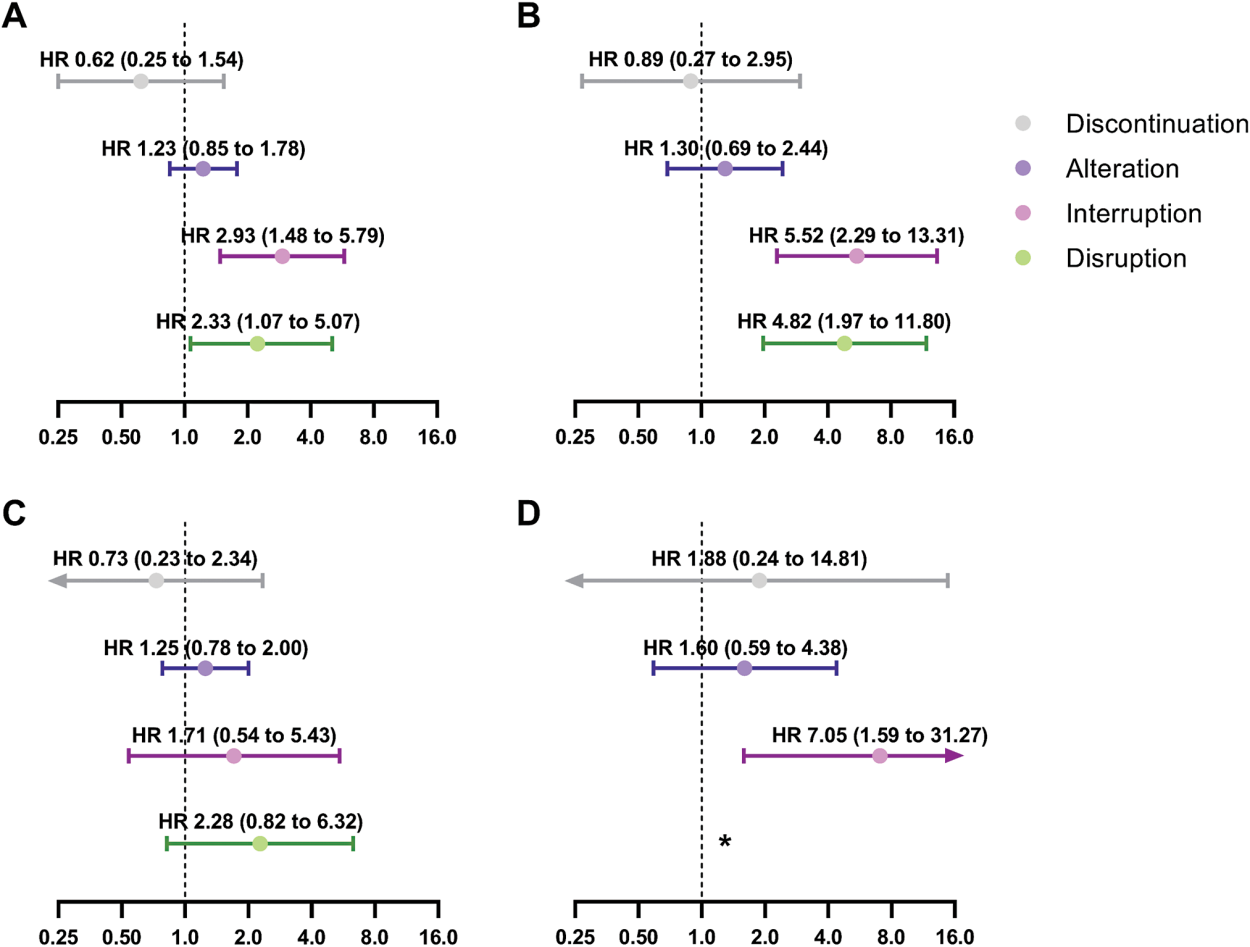
Clinical implications of treatment modification. Values are presented as adjusted hazard ratios (HRs) and corresponding 95% confidence interval (CI) for ( **A** ) ischemic events defined as all-cause death, myocardial infarction, and stroke, ( **B** ) all-cause death, ( **C** ) myocardial infarction, and ( **D** ) stroke. *There were no patients who had a stroke after disruption, and therefore, no HR could be calculated.

The overall incidence of ischemic events was 6.6% ( *n* = 284) at 12 months of follow-up. Incidences of the individual components of the primary ischemic endpoint were 1.9% ( *n* = 81) for all-cause death, 4.2% ( *n* = 181) for MI, and 0.9% ( *n* = 38) for stroke. The incidence of cardiovascular death was 1.0% ( *n* = 43). In total, 57 out of 284 events (20.1% of all events) occurred after one or more treatment modification, so most events (79.9% of all events) occurred while patients were still on uninterrupted ticagrelor therapy. Estimated risk associations for the different types of treatment modification are shown in Fig. 4 . Physician-recommended discontinuation (adjusted HR: 0.62, 95% CI: 0.25–1.54, *p* = 0.31) and alteration (adjusted HR: 1.23, 95% CI: 0.85–1.78, *p* = 0.26) were not associated with a difference in ischemic events. Conversely, interruption was associated with an increased risk of ischemic events (adjusted HR: 2.93, 95% CI: 1.48–5.79, *p* < 0.01). Similarly, disruption was also associated with an increased risk of ischemic events (adjusted HR: 2.33, 95% CI: 1.07–5.07, *p* = 0.03).

## Discussion

The most important findings of the study are as follows: (1) treatment modifications are common within 12 months of follow-up, especially physician-recommended discontinuation and alteration, (2) reasons for treatment modification depend on the type of modification, and (3) treatment interruption and disruption are associated with excess cardiovascular risk. These findings reaffirm the clinical relevance of treatment modifications in patients discharged on ticagrelor and provide novel insights into the interaction between type of modification and cardiovascular risk.

In line with previous studies, our study demonstrated that treatment modifications are common within the first 12 months following hospital admission for ACS. Pooled data from randomized controlled trials evaluating the efficacy and safety of ticagrelor showed premature discontinuation or nonadherence in up to 25% of the 66,870 included patients. 11 Commonly cited reasons for discontinuation or nonadherence were bleeding and dyspnea, but the exact type and implications of treatment modification were not specified. In the PARIS registry, which enrolled 5,031 patients undergoing PCI between 2009 and 2010, the rates of physician-recommended discontinuation, interruption, and disruption were 11.5, 4.6, and 9.8% at 12 months of follow-up. Notably, the rate of discontinuation was much lower compared with the rate in our cohort (26.7% at 12 months), even though more than half of all patients in the PARIS registry presented with chronic coronary syndrome instead of ACS. Possibly, physicians continued DAPT due to concerns of late and very late stent thrombosis, which occurred more often in the era of first-generation drug-eluting stents. 12 13 However, in the PARIS registry discontinuation was associated with a significantly lower rate of major adverse cardiovascular events defined as the composite of cardiac death, definite or probable stent thrombosis, MI, or target lesion revascularization. 6 Importantly, the PARIS investigators argued that this does not imply causal inference between physician-recommended discontinuation and subsequent cardiac risk. This phenomenon is more likely due to appropriate discontinuation of antiplatelet therapy in patients at relatively low risk of ischemic events, which is supported by our finding that the percentage of patients with predicted low ischemic risk was lower in patients who discontinued ticagrelor within 12 months compared with patients without treatment modification. In our study, most patients discontinued ticagrelor (almost) 12 months after initial hospital admission and discontinuation was not associated with an increase (or reduction) in ischemic risk.

In the Treatment With ADP Receptor Inhibitors: Longitudinal Assessment of Treatment Patterns and Events After Acute Coronary Syndrome (TRANSLATE-ACS) registry, 28.3% of patients discharged on ticagrelor after PCI for MI switched to clopidogrel (87.5%) or prasugrel (12.5%) after a median of 50 days. 14 In contrast to our findings, the most cited reason for treatment alteration was socioeconomic (i.e., out-of-pocket costs). The incidence of ischemic events in the 30 days after treatment alteration was low, but only 226 out of 8,672 patients included in the TRANSLATE-ACS registry were discharged on ticagrelor, limiting statistical power to examine the association between alteration and ischemic events. Interestingly, most patients in our cohort were switched to clopidogrel and not to prasugrel, even though bleeding was a relatively infrequent reason for alteration. Prasugrel has a potent inhibitory effect on platelet aggregation and therefore is an alternative to ticagrelor without concerns of a trade-off in efficacy. 15 Pharmacodynamic studies have suggested that switching from ticagrelor to clopidogrel is associated with an increase in platelet reactivity, but this has not translated into an increased ischemic risk in studies examining treatment alteration or de-escalation usually after a short period of ticagrelor therapy. 16 In fact, some studies have even suggested that planned guided (i.e., based on CYP2C19 genotyping) or unguided de-escalation from ticagrelor to clopidogrel is noninferior to standard treatment with ticagrelor with respect to ischemic events and results in a lower incidence of bleeding. 17 18 However, whether this should be the preferred strategy for patients requiring unplanned (e.g., due to side-effects) alteration remains unclear. In general, it is important to counsel patients regarding common side-effects of ticagrelor both at time of discharge and during follow-up. 11 More specifically, patients should be reassured that ticagrelor-induced dyspnea is not associated with compromised cardiac or pulmonary function. 5

Temporary treatment interruption was almost always due to surgical necessity in our study. Previous studies have reported that 4 to 9% of patients undergo noncardiac surgery within 12 months of PCI and/or ACS hospitalization. 19 20 These patients have an increased risk of ischemic events following surgery compared with patients without underlying cardiovascular disease. 21 A comprehensive meta-analysis of observational studies including over 50,000 patients indicated that interruption of antiplatelet therapy before noncardiac surgery reduced the risk of reoperation for major bleeding by more than 50%, but interruption of antiplatelet therapy is also an important predictor of ischemic events following surgery. 22 A retrospective single-center study previously reported that 2.7% of patients interrupt or discontinue ticagrelor due to planned major surgery, which is in line with the incidence (2.8% at 12 months) observed in our cohort. 23 However, observational studies have yielded conflicting results in terms of the excess cardiovascular risk associated with treatment interruption. For example, in the PARIS registry, there was no association between interruption and subsequent ischemic events. Importantly, in the PARIS registry more than half of all interruptions happened between 12 and 24 months when the risk of ischemic events, especially stent-related events, is relatively low. In our study, interruption was associated with an almost 3-fold increase in ischemic risk at 12 months of follow-up. Our findings therefore support current guideline recommendations to delay elective surgery until 12 months after ACS if possible. 24

Disruption due to bleeding or noncompliance has previously been associated to a substantial increase in ischemic risk. Previous studies have demonstrated that this increased risk is highest directly following treatment disruption and attenuates over time. 6 It was not possible to assess the temporal effects of disruption in our cohort due to the limited number of events. Given that bleeding was the most important reason for disruption, strategies to mitigate bleeding are of the utmost importance. This includes early identification of patients at high bleeding risk, routine consideration of proton pump inhibitor therapy and minimal use of bleeding prone drugs, such as nonsteroidal anti-inflammatory drugs. 25

In recent years, P2Y _12_ -inhibitor monotherapy after a short course of DAPT has emerged as an promising treatment strategy following ACS. 26 Ticagrelor seems to be the agent of choice in most cases, due to its reliable effect on platelet reactivity and its predominant use in clinical trials evaluating P2Y _12_ -inhibitor monotherapy. 27 Future studies will need to address what the clinical impact of treatment modifications are in patients treated with P2Y _12_ -inhibitor monotherapy instead of DAPT.

### Limitations

This study has several important limitations. First, 2.5% of patients were lost to follow-up. In theory, this could have resulted in selection bias. However, this group only forms a small proportion of the total study population. Second, treatment modifications were primarily self-reported and therefore subjected to recall bias, although electronic health records and pharmacy prescription logs were also used to corroborate information regarding treatment modification. Third, a statistically significant association between several types of treatment modifications and clinical outcomes as seen in this study does not imply causality. For example, it is unclear if the excess risk associated with interruption or disruption can be attributed to these treatment modifications or (at least in part) should be attributed to other factors, such as comorbidities or procedural factors (e.g., for patients undergoing surgery). Finally, patients included in the present study were exclusively treated with ticagrelor; therefore, results should not be generalized to patients treated with other P2Y _12_ -inhibitors.

## Conclusion

In clinical practice, treatment modifications in ACS patients discharged on ticagrelor are common, although type and reasons for modification are heterogeneous. Treatment interruption and disruption are associated with excess cardiovascular risk and, although causality cannot be implied from our study, these types of modification should preferably only occur under strict supervision or be avoided altogether.

## Notes

**What is known about this Topic?**

- Patients presenting with acute coronary syndrome are frequently treated with potent P2Y _12_ -inhibitors, such as ticagrelor.
- Some patients require treatment modification within 12 months, but clinical reasons and underlying context for treatment modification are heterogeneous and it is unclear what, if any, excess cardiovascular risk can be attributed to these modifications.

**What does this Paper Add?**

- Treatment modifications in acute coronary syndrome patients discharged on ticagrelor are common within 12 months of follow-up, especially physician recommended discontinuation and alteration.
- Treatment interruption and disruption are associated with excess cardiovascular risk and should therefore only occur under strict supervision or be avoided altogether.
